# Analysis of the characteristics and the degree of pragmatism exhibited by pragmatic-labelled trials of antineoplastic treatments

**DOI:** 10.1186/s12874-023-01975-9

**Published:** 2023-06-24

**Authors:** Robbe Saesen, Kevin Depreytere, Karyna Krupianskaya, Joël Langeweg, Julie Verheecke, Denis Lacombe, Isabelle Huys

**Affiliations:** 1 Clinical Pharmacology and Pharmacotherapy Research Unit, Department of Pharmaceutical and Pharmacological Sciences, KU Leuven, Leuven, Belgium; 2 European Organisation for Research and Treatment of Cancer (EORTC), Avenue E. Mounier 83, 1200 Brussels, Belgium

**Keywords:** Pragmatic trials, Explanatory trials, Randomized controlled trials, Oncology, Cancer, Neoplasms, Treatments, Efficacy-effectiveness gap, Real-world evidence, PRECIS-2

## Abstract

**Background:**

Pragmatic clinical trials (PCTs) are designed to reflect how an investigational treatment would be applied in clinical practice. As such, unlike their explanatory counterparts, they measure therapeutic effectiveness and are capable of generating high-quality real-world evidence. However, the conduct of PCTs remains extremely rare. The scarcity of such studies has contributed to the emergence of the efficacy-effectiveness gap and has led to calls for launching more of them, including in the field of oncology. This analysis aimed to identify self-labelled pragmatic trials of antineoplastic interventions and to evaluate whether their use of this label was justified.

**Methods:**

We searched PubMed® and Embase® for publications corresponding with studies that investigated antitumor therapies and that were tagged as pragmatic in their titles, abstracts and/or index terms. Subsequently, we consulted all available source documents for the included trials and extracted relevant information from them. The data collected were then used to appraise the degree of pragmatism displayed by the PCTs with the help of the validated PRECIS-2 tool.

**Results:**

The literature search returned 803 unique records, of which 46 were retained upon conclusion of the screening process. This ultimately resulted in the identification of 42 distinct trials that carried the ‘pragmatic’ label. These studies examined eight different categories of neoplasms and were mostly randomized, open-label, multicentric, single-country trials sponsored by non-commercial parties. On a scale of one (very explanatory) to five (very pragmatic), the median PCT had a PRECIS-2 score per domain of 3.13 (interquartile range: 2.57–3.53). The most and least pragmatic studies in the sample had a score of 4.44 and 1.57, respectively. Only a minority of trials were described in sufficient detail to allow them to be graded across all domains of the PRECIS-2 instrument. Many of the studies examined also had features that arguably precluded them from being pragmatic altogether, such as being monocentric or placebo-controlled in nature.

**Conclusion:**

PCTs of antineoplastic treatments are generally no more pragmatic than they are explanatory.

**Supplementary Information:**

The online version contains supplementary material available at 10.1186/s12874-023-01975-9.

## Background

Randomized controlled trials (RCTs) are considered the bedrock of evidence-based medicine [1]. Their reliance on randomization as a core methodological principle renders them invaluable for assessing the benefits and risks of experimental therapeutic approaches, it has been argued [2]. Both individually and collectively, these studies generate data that shape clinical guidelines in many fields, including oncology. Although the first cancer RCTs were initiated by academic institutions, the growing complexity of the legal and ethical framework which governs the conduct of clinical trials has made it challenging to set up such studies with limited resources, resulting in the gradual expansion of the pharmaceutical industry’s role in developing antitumor medicines [3]. Demonstrating this shift towards the for-profit development of anticancer agents, a recent analysis showed that approximately 90% of RCTs which investigated systemic treatments against common cancers and whose results were published in major scientific journals in the past decade had been either fully or partially funded by companies [4].

The increasingly commercial nature of clinical research in oncology has been accompanied by a rise in the use of surrogate endpoints [4], which for the most part lack validation as predictors of overall survival or quality of life [5–7]. Moreover, trials undertaken by manufacturers to support the regulatory approval and market entry of antineoplastic drugs often feature comparator arms deviating from the standard of care [8–10] and recruit patients on the basis of stringent eligibility criteria that reduce the external validity of their findings [11–13]. Despite their inherent limitations, studies that incorporate elements like these into their design offer important insights into the efficacy of investigational cancer therapies, i.e. their effects measured under ideal circumstances, when they are applied in highly controlled and artificial environments [14–16]. Such trials, which are known as explanatory RCTs [17], address the question of whether an intervention *can* work, regardless of any external factors that could influence the treatment outcome [14, 18]. They are of particular interest to industry sponsors since they can present a product in the best possible light, and because regulators demand them as part of the requirements that need to be satisfied in order to obtain a marketing authorization [19].

Conversely, so-called pragmatic RCTs [17] investigate whether an intervention *will* actually work, taking into account additional aspects that could have an impact on the therapeutic response [14, 18]. As such, they provide clarity on the effectiveness of anticancer medicines, i.e. their effects assessed under real-life conditions, when they are employed in the clinic [14–16]. These studies are characterized by their patient-centered outcome measures, their clinically relevant comparator arms, their real-world settings, their routine follow-up schedules, and their inclusive recruitment of participants, bringing about heterogeneous samples [18, 20, 21]. Pragmatic RCTs produce valuable insights for patients and clinicians by filling existing evidence gaps and contributing to the realization of a learning health system [22]. Moreover, such trials are of great value to payers and healthcare policymakers as they can directly inform health technology assessment (HTA) and reimbursement-related decision-making [23, 24]. Nevertheless, studies of this nature are infrequently performed, judging by the number of self-labelled pragmatic trials reported in the literature [25–27]. According to one estimate [26], they represented fewer than 1% of all RCTs carried out between 1990 and 2010. Pharmaceutical companies are reluctant to conduct them due to their associated business risks, their omission from the list of studies that are standardly imposed on manufacturers, and the paucity of regulatory guidance on how they should be run, among other reasons [19, 20, 28]. Consequently, pragmatic RCTs have so far mainly been undertaken independently from commercial interests [28], using public funding when available [23, 29, 30].

The dominance of explanatory RCTs has contributed to the emergence of what has come to be known as the efficacy-effectiveness gap, i.e. the phenomenon where significant disparities may be seen between a treatment’s efficacy as recorded in pre-approval trials and its effectiveness as observed in clinical practice [31]. In the field of oncology, where the discrepancy between the survival rates of study participants and real-life patients receiving the same therapies can be large, this gap manifests itself particularly clearly [32–36]. Regulators are cognizant of its existence, but they have also dismissed calls for requiring marketing authorization applicants to submit data from pragmatic trials as part of their product dossiers, fearing that this would introduce more uncertainty into the evaluation of the application and lead to the rejection of potentially useful agents [37]. Nonetheless, such studies, being considered sources of high-quality real-world evidence [38, 39], are accepted by regulatory authorities if they allow sponsors to deliver on their post-approval commitments [39, 40]. Bridging the efficacy-effectiveness gap may necessitate an overhaul of the current medicines development paradigm and the creation of new models of partnership between the various stakeholders involved in the process of getting a drug from the bench to the bedside [41–44].

The distinction between pragmatic and explanatory RCTs is not always apparent: many trials exhibit characteristics of both [18]. In reality, clinical studies exist on a spectrum, ranging from fully pragmatic to fully explanatory, with most trials positioning themselves somewhere in between the two ends [18]. To determine an RCT’s place on this continuum, an instrument was developed that enables researchers to evaluate the degree of pragmatism displayed by the study, namely the Pragmatic Explanatory Continuum Indicator Summary (PRECIS) tool [18]. This tool, which was revised after its original publication and is referred to as PRECIS-2 in its current version [21], makes its users appraise the trial in question across nine distinct domains (Table 1), each of which is rated on a scale from 1 (i.e. very explanatory) to 5 (i.e. very pragmatic). The cumulative score awarded then reflects the extent to which the study integrates facets of pragmatism into its design. PRECIS-2, which unlike its predecessor has been validated [45], is in principle intended to be applied prospectively by investigators, when they are writing a trial protocol, so that they are aware of the impact of the study’s setup on the generalizability of the results they will eventually acquire [18, 21]. However, the instrument can also be employed retrospectively, to grade pragmatic RCTs which have already been completed, in the context of a systematic review [46].

**Table 1:** Overview of the nine domains of the PRECIS-2 instrument, their descriptions, and the corresponding questions that users of the tool need to ask themselves when evaluating the degree of pragmatism exhibited by a particular trial [21, 47]

PRECIS-2 domain	Description	Question
Eligibility	The extent to which the trial is open for recruitment to patients with varying demographic characteristics and medical histories	To what extent do the participants of the trial resemble the patients that would receive the intervention in clinical practice?
Recruitment	The amount of effort that is expended to enroll new participants onto the trial	How much effort is made to recruit participants for the study, going beyond what can be considered a standard level of interaction between patients and their doctor?
Setting	The environment in which the trial takes place	To what extent does the environment in which the study is conducted mirror the usual care setting?
Organization	The resources and the expertise that are required to deliver the intervention	To what extent are the resources and the expertise needed to deliver the intervention different from those that would be available in the clinic?
Flexibility—delivery	The degree of flexibility that is permitted with respect to the delivery of the intervention by investigators	How much discretion is granted to the investigators in terms of their administration of the intervention, as compared with the usual care setting in which doctors may or may not choose to prescribe the standard treatment schedule?
Flexibility—adherence	The level of flexibility that is allowed with regard to the compliance of trial participants with the investigational treatment schedule	How much discretion is granted to the participants in terms of their adherence to the intervention, as compared with the usual care setting in which patients may or may not follow the treatment schedule that their doctor prescribes them?
Follow-up	The intensity with which trial participants are monitored and followed up, and the amount of data that are collected during the follow-up period	How intensively are the participants of the study followed up, going beyond what can be considered a standard monitoring and data collection schedule?
Primary outcome	The nature of the endpoint that the trial was statistically designed to measure	To what extent is the primary outcome measure relevant for patients?
Primary analysis	The nature of the method by which the primary outcome data are analyzed statistically	To what extent are all the primary outcome data that were collected for the study included in the analysis?

It has been noted that many self-described pragmatic trials of pharmacological interventions are placebo-controlled and/or double-blinded studies, sometimes performed in a single institution or hospital, examining substances that have not yet been licensed [25]. Given that these features inherently limit the real-life applicability of a trial’s findings, this observation suggests that the ‘pragmatic’ label is often misused, likely because claims of pragmatism can attract attention from readers [25, 48, 49]. Moreover, RCTs that call themselves pragmatic do not commonly explain why they deserve to be designated as such, and the use of the PRECIS-2 tool for this purpose remains extremely rare [50]. Some authors have advocated the conduct of pragmatic cancer trials to address the efficacy-effectiveness gap in oncology [41, 42, 44, 51–54], but it remains unclear what the current landscape of these studies looks like. In this article, we identify clinical trials from the literature that investigated antineoplastic treatments and that were tagged as pragmatic at least once. Subsequently, we assess whether they were justifiably labelled as such by applying the PRECIS-2 instrument.

## Methods

### Literature screening and study selection

We searched PubMed® and Embase® for publications which outlined the design and/or the results of clinical trials of antineoplastic interventions and whose titles, abstracts or index terms made reference to the pragmatic nature of the studies in question. To this end, a search strategy was developed which contained synonyms and variations of the terms ‘pragmatic clinical trial’ (hereinafter abbreviated as PCT) and ‘neoplasm’, including the corresponding MeSH® and Emtree® descriptors (Additional File 1). The list of PubMed® and Embase® entries that were retrieved on 3 February 2021 using this strategy was imported into EndNote® for removal of duplicates. After all duplicates were eliminated, the shortened list was uploaded to Rayyan®, a website which was designed to facilitate the process of reviewing scientific literature in a systematic manner. The titles and abstracts of the items catalogued in Rayyan® were screened by a team of five researchers (RS, KD, KK, JL, JV), with each individual item being examined by three different persons (half of the items by RS, KD and JV, and the other half by RS, KK and JL). Based on various inclusion and exclusion criteria that were defined a priori (Table 2), the researchers selected the publications that they thought should be scrutinized further, without being able to see each other’s selections. Once they had gone through the items in Rayyan® that were assigned to them, the researchers discussed their choices amongst each other (in two groups of three) and any disagreements were resolved through consensus. This procedure was repeated for the full text screening, ultimately leading to the identification of a number of distinct trials carrying the ‘pragmatic’ label.

**Table 2:** Overview of the inclusion and exclusion criteria applied during the literature screening and study selection process

Inclusion criteria	Exclusion criteria
• Publications (full articles or conference abstracts) discussing the design and/or the results of clinical trials • Publications discussing clinical trials that investigate antineoplastic treatments • Publications discussing clinical trials that are described as pragmatic in the title, abstract or index terms	• Publications discussing other types of studies than clinical trials (e.g. cohort studies) • Publications discussing clinical trials conducted in other fields than oncology • Publications written in other languages than English • Publications that are not accessible • Publications discussing clinical trials that investigate non-therapeutic interventions (e.g. screening programs) • Publications discussing clinical trials that investigate complementary therapies (e.g. acupuncture) • Publications discussing clinical trials that investigate symptomatic therapies (e.g. analgesics)

### Data extraction and PRECIS-2 scoring

Next, an in-depth analysis of the identified PCTs was undertaken by consulting publicly accessible sources of information about these studies, such as journal articles, conference posters and abstracts, records from ClinicalTrials.gov and other trial registries, study protocols, and dedicated trial websites, if available. These source materials were again reviewed by five researchers (RS, KD, KK, JL, JV), following the same method that was employed during the literature screening phases. The researchers collected specific data on the studies (Table 3) by filling in a standardized data extraction form. Simultaneously, they applied the PRECIS-2 tool according to the instructions provided by its creators [21, 46, 47] and graded the trials across the nine domains that this instrument encompasses, documenting the motivation behind each score given by quoting relevant excerpts from the source materials. If a domain could not be appraised due to a lack of information found, it was left blank and the trial-level score per domain was determined by adjusting the denominator to reflect the number of assessable domains (e.g. if two domains could not be evaluated and the remaining seven together received a score of 21, the trial-level score per domain would be 21/7 = 3). The researchers were blinded to each other’s data extraction forms and PRECIS-2 scores until they had all completed their review of the source materials. Eventually, they met to compare their forms and scores and to settle any conflicting interpretations by mutual agreement.

**Table 3:** Overview of the characteristics that were recorded for each trial included in the analysis, grouped by category

Category	Characteristic
General aspects	Type(s) of neoplasm(s)
	Year in which the trial was started^♦^
	Year in which the trial was completed^♦^
	Study duration^∟^
	Year in which the main results were published^*^
	Journal in which the main results were published^*^
	Impact factor of journal in which the main results were published^*°^
	Digital object identifier of consulted publication(s)
	Trial registry identifier(s)
	Availability of protocol
Design elements	Clinical phase^▲^
	Use of randomization
	Use of blinding^◊^
	Number of study arms
	Intervention(s)
	Type of intervention(s) (e.g. pharmacotherapy, surgery, radiotherapy, etc.)
	Comparator(s)
	Type of comparator(s) (active or placebo; standard of care or not)^∩^
	Primary endpoint(s)/outcome measure(s)
	Secondary endpoint(s)/outcome measure(s)
Organizational facets	Number of participants^†^
	Site-level setting (monocentric or multicentric)^∆^
	Country-level setting (national or international)^■^
	Countries in which the trial took place
	Legal sponsorship^□^
	Funding source (commercial or non-commercial/academic)^±^

^♦^Information on when the studies were initiated and finalized was obtained from the trial registry records. If such data were missing from these files, they were extracted from other sources (e.g. journal articles)

(∟) Calculated by subtracting the year in which the trial was started from the year in which it was completed

^*^If the main results of the study had not (yet) been published, these characteristics were instead documented for publications discussing the interim results of the trial or, absent any such articles or abstracts, its setup

^°^Impact factors were taken from Web of Science® journal metrics. If the 2021 impact factor was unavailable, the 2020 one was used as a substitute

^▲^Note that the clinical phase concept is not applicable to studies primarily investigating any non-pharmacological interventions other than gene therapies

^◊^A trial was considered single-blinded if either the patients participating in the study or the healthcare professionals administering the intervention were unaware of the treatment allocation, and double-blinded if both parties were. Masking on the part of outcome assessors and data analysts was left out of consideration

^∩^The assessment of whether or not a specific comparator treatment constituted standard of care was based on how it was described in the source materials that were scrutinized for each trial

^†^This characteristic reflects the number of participants that were randomized or allocated to receive a study treatment. For ongoing trials that are still recruiting patients, it represents the planned sample size

^∆^This characteristic denotes whether the study was conducted in a single center or across multiple centers

^■^This characteristic indicates whether the trial was rolled out in a single country or across multiple countries

^□^Information on the legal sponsorship of the studies was collected from the trial registry records

^±^A trial was deemed to have a commercial funding source if it received material or financial support from companies

### Data analysis and reporting

Once the separate data extraction forms had been merged into one and the PRECIS-2 scores had been agreed upon by the researchers, the data gathered were analyzed descriptively in Excel® and inferentially using IBM® SPSS® Statistics 28.0. For nominal variables (e.g. type of neoplasm, primary endpoint, comparator treatment, etc.), proportions were calculated. Ordinal (e.g. sample-level PRECIS-2 scores per domain), discrete (e.g. number of participants, study duration, number of assessable PRECIS-2 domains, etc.) and continuous (e.g. journal impact factor, trial-level PRECIS-2 scores per domain) variables were characterized by their median values, with the associated interquartile ranges (IQRs) being represented by their lower and upper boundaries. Mann–Whitney U tests were performed to verify whether trials with particular features displayed a PRECIS-2 score per domain that was significantly different from that of the studies that did not have those features. To probe the strength of the correlation between the sample size of the trials and their PRECIS-2 score per domain, Kendall’s tau-b coefficient was computed. The significance threshold was set at 0.05.

## Results

### Literature screening and study selection

**Fig. 1:**
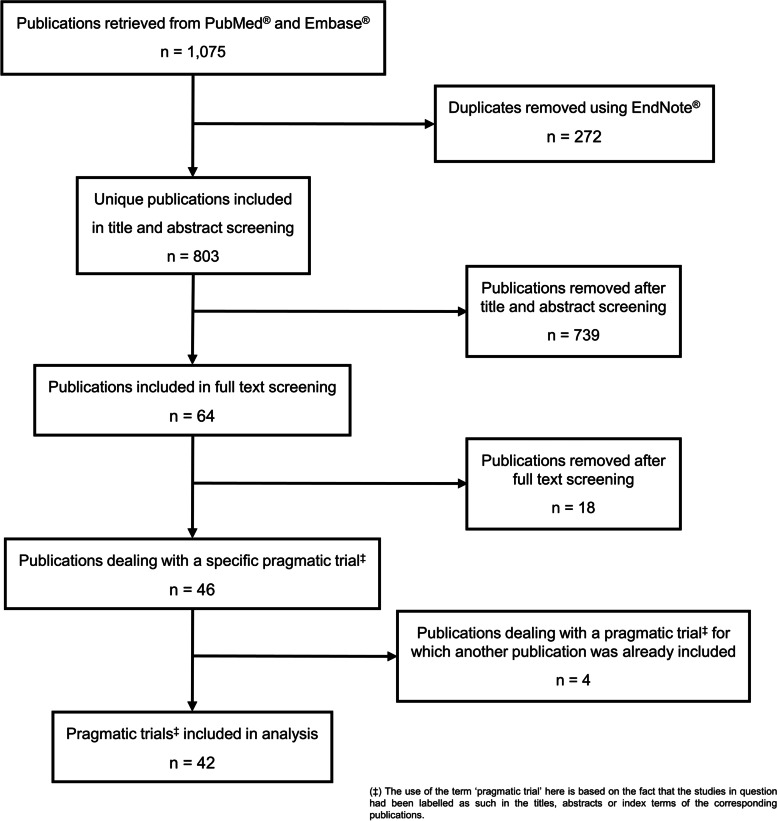
Flowchart of the literature screening and study selection process

The search strategy returned 1,075 results in total, of which 473 were obtained from PubMed® and 602 from Embase® (Fig. 1). After 272 duplicates were filtered out in EndNote®, the titles, abstracts and index terms of the remaining 803 publications were screened in Rayyan® for adherence to the prespecified eligibility criteria. 64 items were withheld for the full text screening, which eventually led to the identification of 42 distinct trials that had been tagged as pragmatic at least once, either by the authors themselves or by the PubMed® and Embase® reviewers that manually assign relevant MeSH® and Emtree® descriptors to new entries in these two repositories.

### Characteristics of included trials

The characteristics of the included trials are summarized in Table 4. The completed data extraction form outlining the information collected for each individual trial is provided in Additional File 2.

**Table 4:** Overview of the characteristics of the 42 trials included in the analysis

Characteristic		Total: *n* = 42	
		**n**	**%**
Type of neoplasm	Gastrointestinal	9	21.4
	Breast	9	21.4
	Hematological	6	14.3
	Gynecological	4	9.5
	Lung	3	7.1
	Genitourinary	3	7.1
	Skin	3	7.1
	Head and neck	2	4.8
	Non-specific	3	7.1
Decade of initiation	1980s	2	4.8
	1990s	5	11.9
	2000s	12	28.6
	2010s	23	54.8
Study status	Completed	35	83.3
	Ongoing	7	16.7
Protocol availability	Accessible	23	54.8
	Inaccessible	19	45.2
Clinical phase	I	2	4.8
	I/II	1	2.4
	II	9	21.4
	II/III	1	2.4
	III	7	16.7
	IV	1	2.4
	N/A	21	50.0
Randomization	Randomized	31	73.8
	Non-randomized	5	11.9
	Single-arm	6	14.3
Blinding	Blinded	2	4.8
	Open-label	40	95.2
Number of study arms	1	6	14.3
	2	30	71.4
	3 or more	6	14.3
Type of intervention	Pharmacotherapy	24	57.1
	Surgery	13	31.0
	Radiotherapy	16	38.1
	Other	7	16.7
	Combination	15	35.7
Type of comparator	Active	34	81.0
	Standard of care or equivalent	28	66.7
	Placebo	2	4.8
	None	7	16.7
Primary endpoint(s)	Overall survival	6	14.3
	Quality of life	1	2.4
	Surrogate	26	61.9
Secondary endpoint(s)	Overall survival	26	61.9
	Quality of life	23	54.8
	Surrogate	36	85.7
Site-level setting	Monocentric	11	26.2
	Multicentric	31	73.8
Country-level setting	National	29	69.0
	International	13	31.0
Legal sponsorship	By industry	3	7.1
	By other party	39	92.9
Funding source	Commercial	15	35.7
	Non-commercial or academic	27	64.3
		**Median**	**IQR**
Study duration (in years)		5.5	3–8
Impact factor journal of publication		9.162	4.853–41.316
Number of participants	Phase II trials	68	51–121
	Phase III trials	501	231.5–748
	Overall	161	87.5–538.5

#### General aspects

**Fig. 2:**
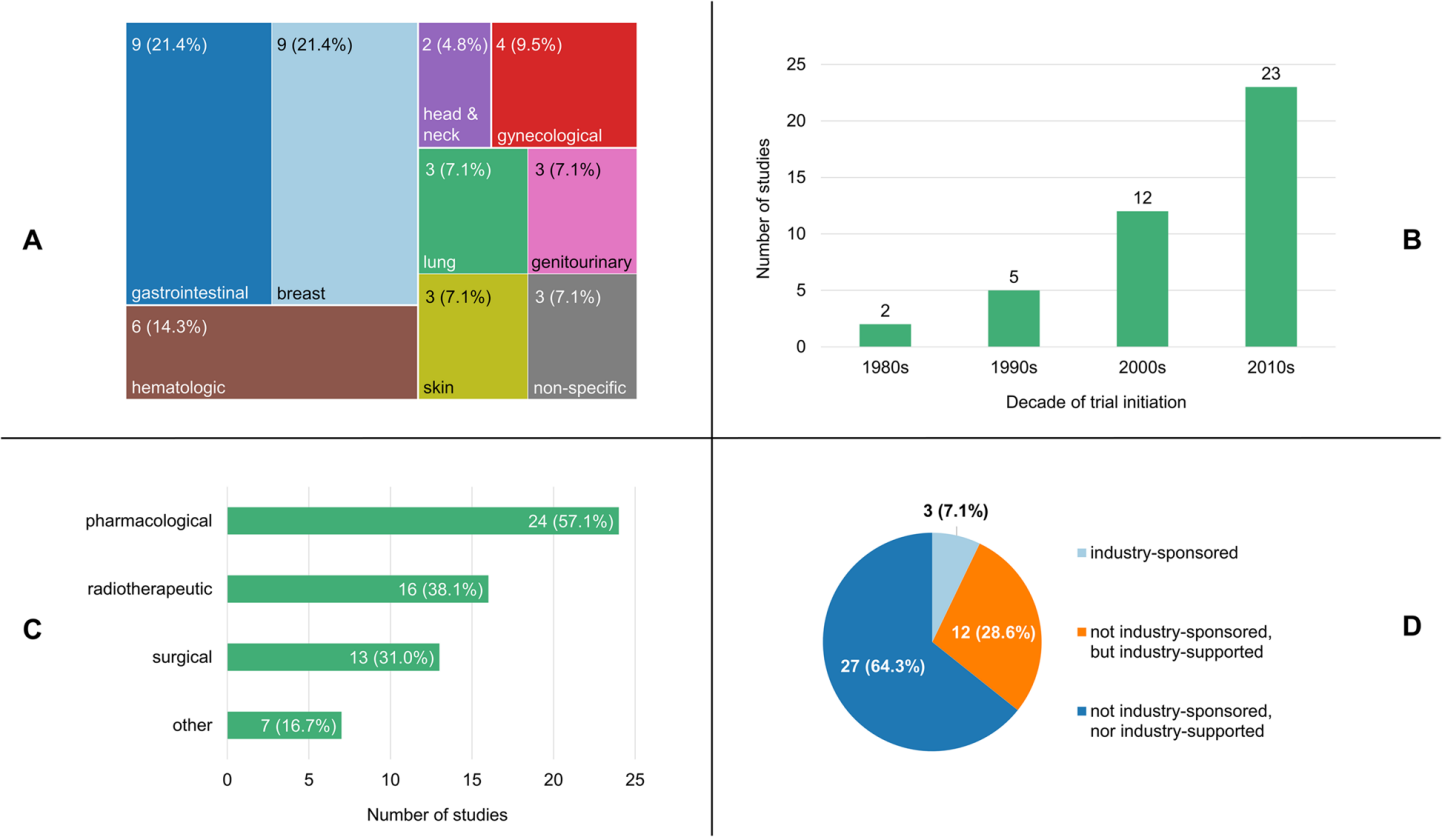
Breakdown of the included studies by (**A**) the types of neoplasms they focused on (with the area of each rectangle/tumor type being proportional to its share of the sample), **B** their decades of initiation, (**C**) the types of interventions they investigated, and (**D**) the extent to which the industry was involved in their conduct. The percentages shown may not add up to 100% due to rounding (**A**), or because the categories displayed are not mutually exclusive (**C**)

The 42 identified PCTs encompassed a wide variety of different neoplasms, which could be categorized into eight distinct groups (Fig. 2A): gastrointestinal neoplasms (9/42, 21.4%), breast neoplasms (9/42, 21.4%), hematological neoplasms (6/42, 14.3%), gynecological neoplasms (4/42, 9.5%), genitourinary neoplasms (3/42, 7.1%), lung neoplasms (3/42, 7.1%), skin neoplasms (3/42, 7.1%), and neoplasms of the head and neck (2/42, 4.8%). Of the three studies that did not focus on a specific tumor type, two dealt with spinal metastases of various primary origins and one was a basket trial. The bulk (23/42, 54.8%) of the PCTs in our sample had been launched in the past decade (Fig. 2B). The oldest study was started in 1982, whereas the most recent one began recruiting participants in 2018. Seven trials were ongoing at the time of writing, and completed PCTs had run for a median of 5.5 years (IQR: 3–8). When results were available for a study (39/42, 92.9%), they had generally been published in high-impact journals (median impact factor of 9.162, with IQR of 4.853–41.316). For most of the PCTs (23/42, 54.8%), the study protocol was publicly accessible, usually as an appendix to a publication detailing the trial’s findings or as a separate article.

#### Design elements

Half (21/42, 50.0%) of the trials included in the analysis covered particular phases of the clinical development paradigm, with phase II (9/21, 42.9%) and phase III (7/21, 33.3%) studies being more prevalent than those of any other phases (I, I/II, II/III, or IV; 5/21, 23.8%). While most (31/42, 73.8%) of the examined PCTs employed randomization and could therefore be considered RCTs, non-randomized and single-arm trials together accounted for more than a quarter (11/42, 26.2%) of the studies scrutinized. The use of masking was exceptional: only two trials (4.8%) had implemented blinding schemes into their design, and in both cases neither the participants nor the investigators were at any point in the data collection process aware of which intervention(s) they had been allocated to receive or administer. All phase III PCTs (7/7, 100%) were randomized, open-label studies.

The sample mainly (30/42, 71.4%) consisted of trials that evaluated two treatment strategies against each other. For a majority (24/42, 57.1%) of the analyzed PCTs, at least one of the study interventions could be classified as pharmacological in nature (e.g. chemotherapy, targeted therapy, hormonal therapy; Fig. 2C). Many trials also investigated therapeutic approaches that comprised surgical (13/42, 31.0%) or radiotherapeutic (16/42, 38.1%) procedures. Over a third (15/42, 35.7%) of the studies explored combinations of different treatment modalities (e.g. intraoperative radiotherapy). If comparator arms were present in the PCTs (35/42, 83.3%), they typically reflected what constituted the standard of care or the most commonly used choice of therapy at the time the trials were initiated (28/35, 80.0%). Placebo controls were exclusively featured in the two blinded studies.

With regard to the outcome measures recorded within the trials, a majority (26/42, 61.9%) of the PCTs had been statistically designed to generate data on one or more surrogate endpoints (e.g. progression-free survival, disease-free survival, response rate, etc.), including all (9/9, 100%) of the phase II and a significant proportion (5/7, 71.4%) of the phase III studies. Overall survival was infrequently (6/42, 14.3%) adopted as a primary endpoint, but often (26/42, 61.9%) served as a secondary one instead. In most (23/42, 54.8%) trials, an assessment of patients’ quality of life was carried out, albeit rarely (1/23, 4.3%) with the intent of delivering on the study’s main objective. Some PCTs (5/42, 11.9%) had been set up to document other types of clinically meaningful outcomes, such as changes in the mobility status or pain response of their participants.

#### Organizational facets

The studies’ sample sizes, whether actual (for completed trials) or planned (for ongoing trials), varied significantly, ranging from 4 to 3,581. The median phase II PCT had 68 participants (IQR: 51–121), whereas the median phase III one had 501 (IQR: 231.5–748). Collectively, the trials that were reviewed in this analysis had a median of 161 patients (IQR: 87.5–538.5) taking part in them, ignoring two prematurely terminated PCTs from which no meaningful conclusions could be drawn, and assuming studies that were still running reached their accrual targets. Approximately three-quarters (31/42, 73.8%) of the trials had been conducted across multiple hospitals or institutions and could thus be described as multicentric. However, only a minority (13/42, 31.0%) of the PCTs had been rolled out in more than a single country. The three countries which saw the most studies being undertaken within their borders were the United Kingdom (16/42, 38.1%), the United States (8/42, 19.0%) and the Netherlands (7/42, 16.7%).

The legal sponsorship of the trials was almost always (39/42, 92.9%) assumed by an entity that did not stand to gain monetarily from their outcomes, such as a university hospital, a research consortium, a learned society, or a not-for-profit organization (Fig. 2D). University College London alone was the sponsor of six (14.3%) of the studies in our sample. The few (3/42, 7.1%) PCTs that were sponsored by companies examined interventions of a commercial nature, namely a type of gene therapy, a topical cream, and a chemotherapeutic agent. Nevertheless, many (12/39, 30.8%) of the trials that were not performed at the industry’s behest still received some degree of financial or material support from the manufacturers of the products they investigated (e.g. educational grants, free drug supplies, etc.).

### PRECIS-2 scores

The completed data extraction form listing the PRECIS-2 scores given to each individual trial is provided in Additional File 2.

For the median PCT, 8 domains of the PRECIS-2 tool could be assessed (IQR: 7–9), resulting in a trial-level score per domain of 3.13 after rounding (IQR: 2.57–3.53). The scores awarded did not depend on whether or not the studies employed randomization (*P* = 0.059), took place in multiple countries (*P* = 0.276), or received support from the industry (*P* = 0.066). Moreover, PCTs for which a protocol was available were not graded significantly higher than those for which no such document could be retrieved (*P* = 0.294). Multicentric trials on the other hand garnered greater scores than their monocentric counterparts (*P* = 0.010).

**Fig. 3:**
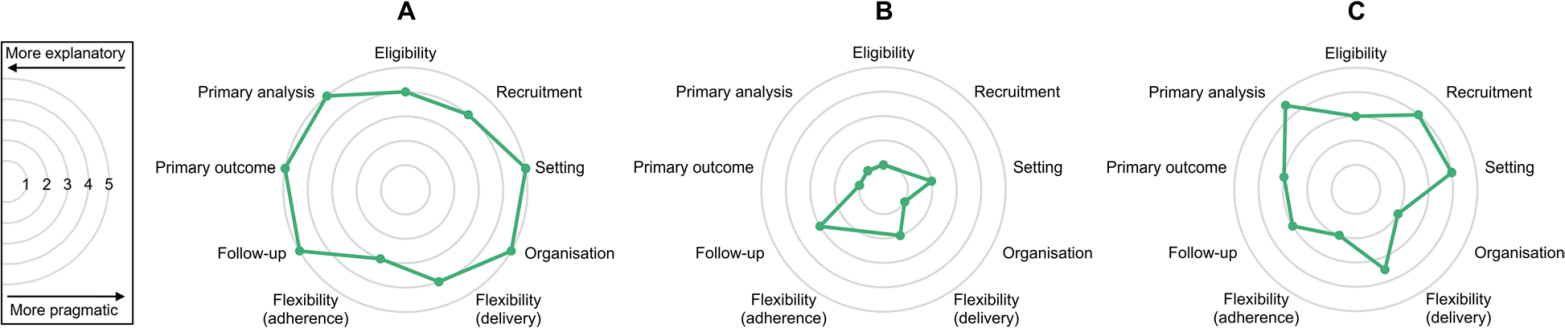
PRECIS-2 wheels of (**A**) the highest-scoring, (**B**) the lowest-scoring, and (**C**) the median trial. The concentric circles represent the different possible scores that can be awarded to each domain of the PRECIS-2 tool, with the innermost one being equivalent to 1 and the outermost one corresponding with 5. The area of the polygons enclosed by the green lines connecting the ‘score dots’ is proportional to the degree of pragmatism exhibited by the studies in question. Note that for (**B**), the ‘recruitment’ and ‘flexibility (adherence)’ domains could not be graded, and the matching dots are therefore missing

A positive correlation of moderate strength was observed between the studies’ sample sizes (planned or actual) and their PRECIS-2 scores (τ_b_ = 0.234, *P* = 0.031). PCTs that were labelled as pragmatic in the index terms of their corresponding publications exclusively (*n* = 9) were rated as more explanatory overall (*P* = 0.023). The most pragmatic study included in the analysis was the UKHAN1 trial [55] (PRECIS-2 score per domain of 4.44, Fig. 3A), while the most explanatory one was the RASHEC RCT [56] (PRECIS-2 score per domain of 1.57, Fig. 3B).

At the sample level, the PRECIS-2 domains that accumulated the highest scores were ‘primary analysis’ (median of 4.5, with IQR of 4–5), ‘recruitment’ (median of 4, with IQR of 3–5) and ‘setting’ (median of 4, with IQR of 3–5), whereas the ones that accrued the lowest were ‘organization’ (median of 2, with IQR of 1–3) and ‘flexibility (adherence)’ (median of 2, with IQR of 2–3). A detailed breakdown of the median PRECIS-2 scores by domain is presented in Table 5 and displayed in Fig. 3C. In most cases (24/42, 57.1%), one or more domains of the PRECIS-2 instrument could not be appraised for a given PCT due to a lack of information provided in the source documents that were consulted for each study. Even when a protocol was accessible (23/42, 54.8%), the appraisal of certain domains was oftentimes (7/23, 30.4%) impossible for this reason. ‘Flexibility (adherence)’ (20/42, 47.6%) and ‘recruitment’ (21/42, 50.0%) were the least frequently assessable domains altogether, with the former sometimes being inapplicable (8/42, 19.0%). Just four domains were scorable across all trials, namely ‘eligibility’, ‘flexibility (delivery)’, ‘primary outcome’ and ‘setting’.

**Table 5:** Overview of the sample-level PRECIS-2 scores per domain. The last column of the table displays the number of times the individual domains could not be graded, either because they were not applicable to the studies in question (e.g. flexibility in terms of adherence cannot be assessed for trials of surgical techniques, as these are single-application interventions), or due to a lack of relevant information provided in the source documents that were consulted for each PCT

PRECIS-2 domain	PRECIS-2 score		Number of times no score could be given
	**Median**	**IQR**	
Eligibility	3	2–4	0
Flexibility (adherence)	2	2–3	22^a^
Flexibility (delivery)	3.5	2–4	0
Follow-up	3	2–4	4
Organization	2	1–3	4
Primary analysis	4.5	4–5	4^b^
Primary outcome	3	2–4	0
Recruitment	4	3–5	21
Setting	4	3–5	0

^a^More specifically, 14 times on account of insufficiently detailed reporting, and 8 times owing to the inapplicability of the domain to the trials at issue

^b^On 3 occasions, this domain was not scorable as a result of it not being pertinent to the studies under evaluation

## Discussion

To our knowledge, this is the first study that has analyzed the characteristics and the degree of pragmatism exhibited by pragmatic-labelled trials of antineoplastic interventions. We found that such trials were heterogeneous in terms of the methodological and organizational features they displayed, and that they were overall only slightly more pragmatic than explanatory in their setup, as evidenced by their moderate PRECIS-2 scores. Additionally, we observed that in most cases, the PCTs could not be graded across all domains of the PRECIS-2 tool because their design and procedures had been inadequately reported in their corresponding publications.

Our analysis shows that the number of pragmatic-labelled studies initiated in the field of oncology has roughly doubled every decade since the 1980s. This rising trend suggests that the use of PCTs to investigate the effectiveness of antitumor therapies has become more popular over time. However, it is debatable whether this actually means that sponsors of trials examining antineoplastic interventions are increasingly embracing the concept of pragmatism, as it could also signify that the ‘pragmatic’ descriptor is being misapplied more frequently. In fact, the PRECIS-2 scores that were obtained seem to support the latter interpretation, and prior literature points in that direction as well [25, 50]. Nevertheless, the expanding role of real-world evidence in the development of anticancer treatments [57, 58] will likely spur the future conduct of PCTs, since these studies are considered to be some of the most robust sources of such evidence [38, 39].

Some authors [59–72] have employed the PRECIS-2 instrument as a critical appraisal tool to investigate the extent to which the trials that they included in their systematic or scoping literature reviews could be classified as PCTs. Others [73–86] have used it in a similar manner to us, with the aim of verifying whether studies that were tagged as pragmatic or that were running as part of a publicly funded PCT program deserved to carry the ‘pragmatic’ label. However, few publications [69, 71, 72] in which PRECIS-2 assessments were undertaken looked at trials of antineoplastic interventions, and only one reported the outcomes of an analysis that was comparable to ours in terms of its sample size and methodological depth. Sorigue and Kuittinen [72] evaluated the position of studies underpinning the therapeutic recommendations listed in the 2020 European Society for Medical Oncology clinical guidelines for follicular lymphoma on the pragmatic-explanatory continuum. In total, they incorporated 28 distinct trials into their review, more than half of which could only be scrutinized based on articles outlining their results due to the inaccessibility of their protocols. The median trial received a PRECIS-2 score per domain of 3.5, which is slightly higher than in our analysis. This is remarkable, given that the studies we examined had been explicitly labelled as pragmatic, whereas the ones appraised by Sorigue and Kuittinen [72] had not. Moreover, the sample-level PRECIS-2 scores per domain differed significantly across the two analyses: while ‘organization’ was for instance the joint-most explanatory domain for our trials, it was among the most pragmatic for those reviewed by Sorigue and Kuittinen [72]. These observations further illustrate how the ‘pragmatic’ label is often used in a misleading manner, offering no assurance that the study in question is more pragmatic than any given trial not carrying this tag. Nevertheless, it should be stressed that a direct comparison between our findings and those of Sorigue and Kuittinen [72] may not be fully appropriate: unlike us, Sorigue and Kuittinen [72] did not consider the ‘flexibility (adherence)’ and ‘recruitment’ domains of the PRECIS-2 instrument at all in their analysis, since they could not find any meaningful information on the basis of which these domains could be graded. Consequently, differences in scoring may be the result of divergence in the way the PRECIS-2 instrument was applied.

Concerning the methodological design of the PCTs in our analysis, two studies employed the so-called trials-within-cohorts (TwiCs) approach [87], namely VERTICAL [88] and RECTAL-BOOST [89]. These studies are therefore examples of cohort multiple randomized controlled trials (cmRCTs) [87, 90, 91]. In such trials, patients are followed prospectively as part of a large observational cohort and a randomly selected subset among them is given the choice to undergo the investigational intervention [87, 90, 91]. Participants who are randomized to remain within the cohort receive usual care and make up the control group of the study. Since these patients are not informed of the fact that a trial is being conducted and that treatment allocation has taken place, the recruitment process can be significantly more efficient and less contrived for cmRCTs than for regular RCTs [90–92]. Moreover, because their cohorts are intrinsically embedded into clinical practice, cmRCTs are standardly performed in a real-world setting [87, 90, 91]. As a result, cmRCTs tend to be very pragmatic with respect to the ‘recruitment’ and ‘setting’ domains of the PRECIS-2 tool. However, as TwiCs schemes are relatively new, investigators may not yet be very familiar with them. Consequently, additional training could be required, lowering the score that can be given for the ‘organization’ domain. Besides cmRCTs, our sample also contained a basket trial [93], namely TAPUR [94]. Here as well, the innovative nature and relative complexity of biomarker-driven studies [93] limit the degree to which they can be pragmatic in terms of their organization. Furthermore, the stringent criteria that such trials apply to determine which drugs should be administered to which patients [93] inherently restrict the level of flexibility that is allowed with regard to the delivery of the targeted agents in question. Nevertheless, basket trials can still be justifiably labelled as pragmatic, as long as they implement enough elements of pragmatism into their design.

Notably, over a quarter of the PCTs that were examined in this analysis could not be regarded as RCTs, either because they were single-arm trials or because their participants were not randomly assigned to their study arms. In principle, a lack of randomization does not necessarily exclude a trial from being pragmatic: in their seminal paper introducing the concept of pragmatism [17], Schwartz and Lellouch never specifically refer to randomized trials, only to trials in general. However, the authors of the PRECIS-2 tool explicitly state that their instrument is intended to be applied to RCTs [21], even though none of its domains directly pertain to the method used for treatment allocation. In fact, it can be argued that randomization is a highly artificial procedure, since in a real-life environment, patients and clinicians can actively choose which interventions they will undergo or administer. Nevertheless, RCT designs are methodologically indispensable for producing robust and actionable evidence which is free from confounding-related biases [2, 22]. Hence, it may be reasonable to consider randomization separately from the PRECIS-2 assessment, for example as part of a critical appraisal exercise based on the Cochrane risk-of-bias tool RoB 2 [95]. By randomizing at the site level rather than the individual participant level, RCTs can preserve their statistical strengths and at the same time emulate real-world practice more closely. This technique, which is referred to as cluster randomization [96], is commonly employed in PCTs [97] but was not featured in any of the studies in our sample.

Despite reviewing all of the available source documents for the included studies, we were unable to appraise most of the PCTs in our sample across every domain of the PRECIS-2 instrument. Other authors [63, 65, 68, 70, 72, 76, 79–81] have likewise struggled to conduct exhaustive PRECIS-2 evaluations within the context of a systematic or scoping literature review. The fields corresponding with the ‘flexibility (adherence)’ and ‘recruitment’ domains on the tool’s accompanying appraisal sheet seem to be especially difficult to complete, even when considering that the former domain is not universally applicable. The inability to fully grade a given trial due to missing information is a known limitation of retrospective PRECIS-2 assessments [98]. Examining the protocol in addition to publications describing the results of the study does not always solve the problem: in our analysis, almost one-third of the trials for which this document could be accessed had at least one outstanding PRECIS-2 domain. Echoing the proposals made by Dal-Ré et al. [25], we believe that PCT sponsors and investigators should (a) systematically and prospectively evaluate the degree of pragmatism exhibited by their studies using the PRECIS-2 tool, and (b) publish the scores given for each domain along with the underlying motivations so that journal editors, reviewers and readers can independently validate them.

None of the studies in our sample that were started after 2009 (i.e. the year in which the first version of the PRECIS instrument was released [18]) substantiated their claim of being pragmatic by reporting their PRECIS or PRECIS-2 scores. Whenever justification for the use of the ‘pragmatic’ label was provided, it mostly addressed just one or two domains of these tools, usually the ones relating to the nature of the eligibility criteria that were applied to select participants for the trial, the setting in which the study took place, or the level of flexibility that was permitted regarding the administration of the investigational intervention. Janiaud et al. [50] have made similar observations: in their review of 73 pragmatic-labelled RCTs whose results were published in 2016, they found that 45% offered no explanation for why they carried that label, and that only one trial publication included a PRECIS assessment. The studies that tried to justify their ‘pragmatic’ tag mainly did so by citing the same type of arguments that we came across in our analysis. There seems to be a general lack of awareness among PCT sponsors and investigators of the PRECIS(-2) instrument’s existence and of the fact that pragmatism is a multifaceted concept, encompassing all aspects of a trial’s design and organization. Additional efforts may be needed to educate these stakeholders on the hallmarks of PCTs. In light of the important role such studies can play in the decision-making of HTA agencies, payers, clinicians and patients, it is imperative that the ‘pragmatic’ descriptor is employed correctly in the literature.

Some authors [25, 98] have argued that certain design elements can preclude a trial from being pragmatic altogether. More specifically, it has been asserted that placebo-controlled, blinded, single-center and/or pre-approval studies can never be considered PCTs, since such characteristics would inherently render them explanatory. In our analysis, we encountered many trials that displayed at least one of these features. However, instead of immediately excluding the possibility that they could be pragmatic, we still subjected them to a full PRECIS-2 evaluation. That way, if a study scored low for one or two domains, it could nonetheless accrue a relatively high total score by exhibiting a greater degree of pragmatism across the other domains of the PRECIS-2 instrument. This approach is consistent with the recommendations formulated by the tool’s developers [46]. Nevertheless, if the core aim of a PCT is indeed to emulate clinical practice, we would agree that having a monocentric setup and making use of masking would prevent that aim from being achievable. Regardless of how the concept of pragmatism is interpreted, it is remarkable that a large number of pragmatic-labelled trials of antitumor treatments show such overt signs of actually being considerably explanatory.

Strengths of the present study include its wide scope, encompassing all types of neoplasms and interventions, its comprehensive review of virtually every document available on the included trials, and its methodological rigor stemming from its blinded and comparative approaches with respect to data extraction and PRECIS-2 scoring. However, this analysis also suffers from a number of limitations. Firstly, our literature search only allowed us to find studies that had been tagged as pragmatic in the titles, abstracts or index terms of their corresponding publications. This implies that we were unable to identify PCTs for which no results or design characteristics had been reported yet or which had not explicitly been labelled as pragmatic. Nevertheless, the former would likely not have been assessable using the PRECIS-2 instrument given the scarcity of accessible information on such trials, and the latter were not considered for inclusion as they would not have contributed to answering the main research question. To compose a detailed overview of the PCT landscape in oncology, a different search strategy would need to be used, such as the one described by Taljaard et al. [99]. Secondly, several domains of the PRECIS-2 tool were challenging to appraise without in-depth knowledge on the way in which usual care is organized for patients with specific tumors. Since none of the researchers involved in the PRECIS-2 assessment had any experience working as a clinician, some facets of clinical practice may have been unknown to them. To familiarize themselves with such aspects, they consulted additional source materials (e.g. journal articles, chapters from medical textbooks, websites targeted towards patients) where necessary. Lastly, our sample size was relatively small, which could have negatively affected the power of the statistical tests that were performed. As a result, we may have mistakenly failed to reject their null hypotheses on multiple occasions.

## Conclusions

Pragmatic-labelled trials of antineoplastic interventions with published results and/or designs varied in their characteristics, but were mainly randomized, open-label, multicentric, single-country studies that were sponsored by non-commercial parties and that compared pharmacological therapies against standard-of-care treatments in terms of their potential to improve outcomes serving as surrogate markers for the survival and/or quality of life of patients with specific tumors. Despite having been tagged as pragmatic, the trials generally did not exhibit a high degree of pragmatism, judging by the relatively low PRECIS-2 scores they accrued. Some of the studies also displayed features that were arguably irreconcilable with their claims of being pragmatic, like having a monocentric setup or a placebo comparator arm. However, most of the trials were not described in enough detail in their corresponding publications to enable them to be appraised across all domains of the PRECIS-2 tool. Sponsors of future PCTs undertaken in the field of oncology and beyond should systematically justify their use of the ‘pragmatic’ label by prospectively evaluating the position of their studies on the pragmatic-explanatory continuum with the help of the PRECIS-2 instrument or its forthcoming successor, and such an evaluation should be independently reviewed and validated.

## Supplementary Information

**Additional file 1.**

**Additional file 2.**

## Data Availability

All data generated or analyzed during this study are included in this published article and its supplementary information files.

## Abbreviations

cmRCT: Cohort multiple randomized controlled trial
HTA: Health technology assessment
IQR: Interquartile range
PCT: Pragmatic clinical trial
PRECIS: PRagmatic Explanatory Continuum Indicator Summary
RCT: Randomized controlled trial
TwiCs: Trials-within-cohorts
