# Supplementary material for: Analysis of the characteristics and the degree of pragmatism exhibited by pragmatic-labelled trials of antineoplastic treatments
Source: BMC Med Res Methodol. 2023 Jun 24;23:148. doi: 10.1186/s12874-023-01975-9 (PMC10290324; doi:10.1186/s12874-023-01975-9)
Supplement: Supplementary file 1 — Additional file 1. [file 12874_2023_1975_MOESM1_ESM.pdf]

Additional File 1: Search strategy used to find published pragmatic-labelled trials.

PubMed:

("Pragmatic Clinical Trials as Topic"[Mesh] OR "Pragmatic Clinical Trial"[Publication Type] OR "Pragmatic Clinical Trial*"[tiab] OR "Pragmatic Trial*"[tiab] OR "Pragmatic Clinical Stud*"[tiab] OR "Pragmatic Stud*"[tiab] OR "Pragmatic Randomized Controlled Trial*"[tiab] OR "Pragmatic Randomised Controlled Trial*"[tiab] OR "Pragmatic RCT*"[tiab] OR "Pragmatic Randomised Trial*"[tiab] OR "Pragmatic Randomized Trial*"[tiab] OR "Pragmatic Randomised Stud*"[tiab] OR "Pragmatic Randomized Stud*"[tiab] OR "Pragmatic Controlled Trial*"[tiab] OR "Pragmatic Controlled Stud*"[tiab] OR "Naturalistic Clinical Trial*"[tiab] OR "Naturalistic Clinical Stud*"[tiab] OR "Naturalistic Stud*"[tiab] OR "Naturalistic Trial*"[tiab] OR "Naturalistic Randomized Controlled Trial*"[tiab] OR "Naturalistic Randomised Controlled Trial*"[tiab] OR "Naturalistic RCT*"[tiab] OR "Naturalistic Randomised Trial*"[tiab] OR "Naturalistic Randomized Trial*"[tiab] OR "Naturalistic Randomised Stud*"[tiab] OR "Naturalistic Randomized Stud*"[tiab] OR "Naturalistic Controlled Trial*"[tiab] OR "Naturalistic Controlled Stud*"[tiab] OR "Practical Clinical Trial*"[tiab] OR "Practical Clinical Stud*"[tiab] OR "Practical Stud*"[tiab] OR "Practical Trial*"[tiab] OR "Practical Randomized Controlled Trial*"[tiab] OR "Practical Randomised Controlled Trial*"[tiab] OR "Practical RCT*"[tiab] OR "Practical Randomised Trial*"[tiab] OR "Practical Randomized Trial*"[tiab] OR "Practical Randomised Stud*"[tiab] OR "Practical Randomized Stud*"[tiab] OR "Practical Controlled Trial*"[tiab] OR "Practical Controlled Stud*"[tiab]) AND ("Neoplasms"[Mesh] OR "Neoplas*"[tiab] OR "Cancer*"[tiab] OR "Malignan*"[tiab] OR "Oncolog*"[tiab] OR "Carcinoma*"[tiab] OR "Tumor*"[tiab] OR "Tumour*"[tiab])

Embase:

(‘Pragmatic Trial’/exp OR ‘Pragmatic Clinical Trial*’:ab,ti OR ‘Pragmatic Trial*’:ab,ti OR ‘Pragmatic Clinical Stud*’:ab,ti OR ‘Pragmatic Stud*’:ab,ti OR ‘Pragmatic Randomized Controlled Trial*’:ab,ti OR ‘Pragmatic Randomised Controlled Trial*’:ab,ti OR ‘Pragmatic RCT*’:ab,ti OR ‘Pragmatic Randomised Trial*’:ab,ti OR ‘Pragmatic Randomized Trial*’:ab,ti OR ‘Pragmatic Randomised Stud*’:ab,ti OR ‘Pragmatic Randomized Stud*’:ab,ti OR ‘Pragmatic Controlled Trial*’:ab,ti OR ‘Pragmatic Controlled Stud*’:ab,ti

OR ‘Naturalistic Clinical Trial*’:ab,ti OR ‘Naturalistic Clinical Stud*’:ab,ti OR ‘Naturalistic Stud*’:ab,ti OR ‘Naturalistic Trial*’:ab,ti OR ‘Naturalistic Randomized Controlled Trial*’:ab,ti OR ‘Naturalistic Randomised Controlled Trial*’:ab,ti OR ‘Naturalistic RCT*’:ab,ti OR ‘Naturalistic Randomised Trial*’:ab,ti OR ‘Naturalistic Randomized Trial*’:ab,ti OR ‘Naturalistic Randomised Stud*’:ab,ti OR ‘Naturalistic Randomized Stud*’:ab,ti OR ‘Naturalistic Controlled Trial*’:ab,ti OR ‘Naturalistic Controlled Stud*’:ab,ti OR ‘Practical Clinical Trial*’:ab,ti OR ‘Practical Clinical Stud*’:ab,ti OR ‘Practical Stud*’:ab,ti OR ‘Practical Trial*’:ab,ti OR ‘Practical Randomized Controlled Trial*’:ab,ti OR ‘Practical Randomised Controlled Trial*’:ab,ti OR ‘Practical RCT*’:ab,ti OR ‘Practical Randomised Trial*’:ab,ti OR ‘Practical Randomized Trial*’:ab,ti OR ‘Practical Randomised Stud*’:ab,ti OR ‘Practical Randomized Stud*’:ab,ti OR ‘Practical Controlled Trial*’:ab,ti OR ‘Practical Controlled Stud*’:ab,ti) AND (‘Neoplasm’/exp OR ‘Oncology’/exp OR ‘Neoplas*’:ab,ti OR ‘Cancer*’:ab,ti OR ‘Malignan*’:ab,ti OR ‘Oncolog*’:ab,ti OR ‘Carcinoma*’:ab,ti OR ‘Tumor*’:ab,ti OR ‘Tumour*’:ab,ti)
